# Multiple Light Scattering Measurements for Online Monitoring of Milk Fermentation

**DOI:** 10.3390/foods10071582

**Published:** 2021-07-07

**Authors:** Mohsen Ramezani, Giovanna Ferrentino, Ksenia Morozova, Matteo Scampicchio

**Affiliations:** Faculty of Science and Technology, Free University of Bozen-Bolzano, Piazza Università 5, 39100 Bolzano, Italy; mohsen.ramezani@unibz.it (M.R.); ksenia.morozova@unibz.it (K.M.); matteo.scampicchio@unibz.it (M.S.)

**Keywords:** yogurt, fermentation, starter concentration, antibiotics, multiple light scattering

## Abstract

The present paper investigates the use of multiple light scattering for the monitoring of milk fermentation. The experiments were performed on milk fermented with different starter concentrations (0.05% to 4.5% (*w/w*) at temperatures from 36 to 44 °C and in the presence of antibiotics at concentrations up to 100 µg/kg. The fermentation was monitored continuously by using a multiple light scattering technique and simultaneously by a pH meter, a rheometer and a texture analyzer. The backscattering signal recorded by multiple light scattering measurements was correlated with the changes in pH, rheological parameters and firmness of the samples along the fermentation. A gelation time of 120 min was obtained when the highest concentration of starter (4.5%, *w/w*) and incubation temperature of 44 °C were used. These results were confirmed by the pH, rheological and texture monitoring. The analysis of backscattering spectra allowed the detection of the effect of antibiotic on the gel formation even at low concentrations (1.3 µg/kg). Overall, the results highlighted the advantages of using a multiple light scattering technique as quality control tool for online monitoring of milk fermentation.

## 1. Introduction

Yogurt is a popular dairy product traditionally made by fermentation of milk using a mixture of thermophilic starter bacteria (i.e., *Streptococcus thermophilus* and *Lactobacillus delbrueckii* subsp. *bulgaricus*) [1,2,3]. During yogurt production, milk is generally heated. Exposure of whey proteins to heat leads to structural changes that can induce the formation of gel networks above a critical protein concentration. Such minimum concentration depends, in turn, from several factors, like pH, ionic strength and the degree of denaturation [4].

The dynamics of yogurt gel formation during fermentation is generally investigated with empirical approaches [5,6]. In most of the cases, the control of the fermentation process is performed by monitoring the pH changes along time [7] while the end point of the fermentation is obtained when the pH reaches a value equal to 4.1. However, at industrial scale, the control of the fermentation of dairy products is difficult to perform by continuously measuring the pH as the probe can be easily contaminated by milk proteins that adhere to its surface, affecting the measurement. This means that the probe needs to be frequently cleaned and recalibrated. This practice is difficult to carry out. Moreover, glass sensors are usually not accepted in food applications because of concerns about possible glass breakage along the processing line.

As an alternative, measurements at laboratory scale implying the sampling of the product during the process can be used. However, these practices are time consuming and can lead to product contaminations. This is the reason why pH, texture or rheological measurements are not so frequently applied. As a consequence, a poor control of the fermentation process is often achieved resulting in products with possible defects.

Over the years, several techniques have been investigated aiming at identifying ways to effectively monitor milk fermentation [8,9,10,11]. Cimander et al. [12] monitored yogurt fermentation using an electronic nose, a near-infrared spectrometer and standard bioreactor probes. The sensor signals were collected using a cascade neural network predicting quantitative and qualitative process variables, gaining insights on the time of coagulation. Compared to other studies, they were able to predict the effect of the process variables. However, the pH prediction during milk fermentation was poor. In a similar vein, Navrátil et al. [13] used a near-infrared spectrometer coupled with an electronic nose for online monitoring of yogurt and filmjolk (a Swedish yogurt-like sour milk) fermentation. The signals were used to set up empirical partial least-squares models for prediction of pH and titratable acidity. The model contained five parameters and reported some difficulties when applied at industrial level. Moreover, it was not able to accurately predict the pH at the final end point of fermentation. Recently, Arango et al. [14] set up a near infrared scatter probe to control milk fermentation. The data were then correlated with the pH evolution through the development of a mathematical model. The approach was promising, although for industrial implementation the model needed a calibration taking into account the specific production conditions of the yogurt plant.

Although the results of these studies are promising, they presented some drawbacks highlighting the need to investigate for more rapid, easy, non-destructive and online monitoring methods as quality control tools for milk fermentation.

Recently, multiple light scattering technique has received increasing attention from the research and industrial communities thanks to its ability to detect physical aggregation phenomena based on the scattering and transmission of light through a sample [15,16,17,18,19,20].

Based on this background, the present work aims to investigate the potential of multiple light scattering techniques for real-time monitoring of milk fermentation. Different conditions of incubation temperatures, starter concentrations and presence of antibiotics were tested to demonstrate its feasibility. The results were further compared with those obtained by rheological, texture and pH measurements.

## 2. Materials and Methods

### 2.1. Materials

Pasteurized whole milk (3.6% fat, 3.3% protein, 5% lactose) was purchased from a local market. To avoid the effect of daily variation of the milk composition, different batches of the product were frozen in 50 mL falcon tubes and stored at −80 °C until further use [21]. A commercial yogurt starter consisting of Streptococcus salivarius subsp. thermophilus and Lactobacillus delbrueckii subsp. bulgaricus, was purchased from Yogurt Linea (Insao Srl, Milan, Italy). Ceftiofur was purchased from Sigma-Aldrich (Milan, Italy).

### 2.2. Samples Preparation

For the experiments, 200 mL of milk were transferred into an Erlenmeyer flask (250 mL) and mixed thoroughly at room temperature with the yogurt starter culture using the following concentrations: 0.05%, 0.5%, 1.5%, 2.5%, 3.5% and 4.5% (*w/w*). The mixing was carried out using an ultraturrax homogenizer (Ultraturrax T25, IKA, Staufen, Germany) for 20 s at 10,000 rpm. To assess the effect of the antibiotic addition, ceftiofur concentrations were prepared corresponding to 10, 20, 30, 40, 50, 75, and 100 µg/kg in milk. The concentrations were chosen based on the value of the maximum residue limit legally tolerated in a food product [22]. The antibiotic stock solution was freshly prepared on the day of the experiments by dissolving 1 mg of ceftiofur in 50 mL of deionized water (0.002% *w/v*).

### 2.3. Multiple Light Scattering Monitoring

A Turbiscan tower (Formulaction, Toulouse, France) was used to monitor online the backscattering of light during milk fermentation. The apparatus comprised six stations for samples and a detection head equipped with a near-infrared light source (880 nm) scanning the length of the sample’s height and acquiring transmission and backscattering spectra every 20 μm. About 18 mL of samples were transferred into cylindrical glass vials (25 mm i.d., 70 mm height). The milk samples were directly incubated in the tower at different temperatures (36, 38, 40, 42 and 44 °C), starter concentrations (0.05%, 0.5%, 1.5%, 2.5%, 3.5% and 4.5%, *w/w*) and ceftiofur concentrations (10, 20, 30, 40, 50, 75, and 100 µg/kg). They were monitored over a period of 1080 min. Each measurement was performed in triplicate. The analyzer was equipped with a pulsed near infrared light source (λ = 880 nm) and synchronous optical detectors which determined the intensity of backscattered light. The following formulas were applied:

(1) $$BS\approx 1/\sqrt{\ell^{*}}$$

where $\ell^{*}$ is the mean free path of a photon in the disperse system, which, in turn, is defined as:

(2) $$\ell^{*}\left(\varphi ,d\right)=\frac{2\cdot d}{3\cdot \varphi \cdot \left(1-g\right)\cdot Q_{s}}$$

where d is the mean diameter of particles, φ is the volume fraction of particles, and *g* and *Qs* are optical parameters given by the Mie theory. In detail, *Qs* is the scattering efficiency factor while *g* is the asymmetry factor, which depends on the anisotropic scattering of light by a particle. Both parameters are function of the particles size and aggregation phenomena occurring in the sample.

### 2.4. Texture Monitoring

The texture measurements were performed by a back extrusion test using a TA.XTPlus C Texture Analyzer (Stable Micro System, Vienna Court, United Kingdom) equipped with a 50 kg load cell. The apparatus employed a rig (model A/BE, Stable Micro Systems) consisting of a flat 45 mm diameter Perspex disc plunger that moved within a 50 mm inner diameter Perspex cylindrical container. To obtain a series of measurements during the fermentation, the milk samples (75 mL) were directly fermented in the Perspex cylindrical containers of the Texture Analyzer. The milk samples were inoculated with 4.5% (*w/w*) of yogurt starter culture and incubated at 44 °C. After the desired time of incubation, the samples were cooled down immediately in an ice bath to reduce the metabolic activity of the yogurt starter culture. The analysis was performed in compression mode. The test was replicated two times using a pretest speed of 1.0 mm·s^−1^, and a test speed of 5.0 mm·s^−1^. The probe was positioned at a distance of 30 mm above the top of the sample, penetrated inside the sample to a depth of 30 mm and returned to the starting position with a posttest speed of 1.0 mm·s^−1^. The maximum force during the compression cycle was indicated as firmness of the sample and was selected to analyze the system.

### 2.5. pH Monitoring

A pH meter (HACH–sensIONTM, Vetrotecnica, Verona, Italy) was used to determine the pH of milk samples during fermentation. About 18 mL were inoculated with 4.5% (*w/w*) starter culture, transferred to glass vials and incubated at 44 °C in a water bath. The pH was measured every 30 min for 300 min. The measurements were performed in triplicate.

### 2.6. Monitoring of Rheological Parameters

The rheological properties of milk inoculated with defined concentrations of starter were determined using a Discovery Hybrid Rheometer HR-2 from TA-Instruments Co (Milano, Italy). An aliquot of 50 mL of milk was prepared with 4.5% (*w/w*) of starter and transferred to a starch cell equipped with a controlled temperature chamber. Accordingly, the temperature of the chamber was set to 44 °C. During fermentation, the rheological properties (storage (G′), and loss (G″) moduli and loss tangent (tan δ)) of yogurt were acquired by small-amplitude, continuous oscillation measurements. The sample surface in the starch cell was covered with a vegetable oil to prevent evaporation. During fermentation, the sample was oscillated at a frequency of 0.1 Hz. An applied strain of 1% was used to avoid the rupture of the network structure during fermentation. Data were acquired every 375 s for 500 min. The experiments were performed in triplicate.

### 2.7. Statistical Analysis

The software XLSTAT (Version 2016.02.28014, Addinsoft, New York, NY, USA) was used to statistically evaluate the results by applying the analysis of variance (ANOVA). The significant differences between values were analyzed by the Tukey test.

## 3. Results

### 3.1. Monitoring of Milk Fermentation by Multiple Light Scattering

**Figure 1:**
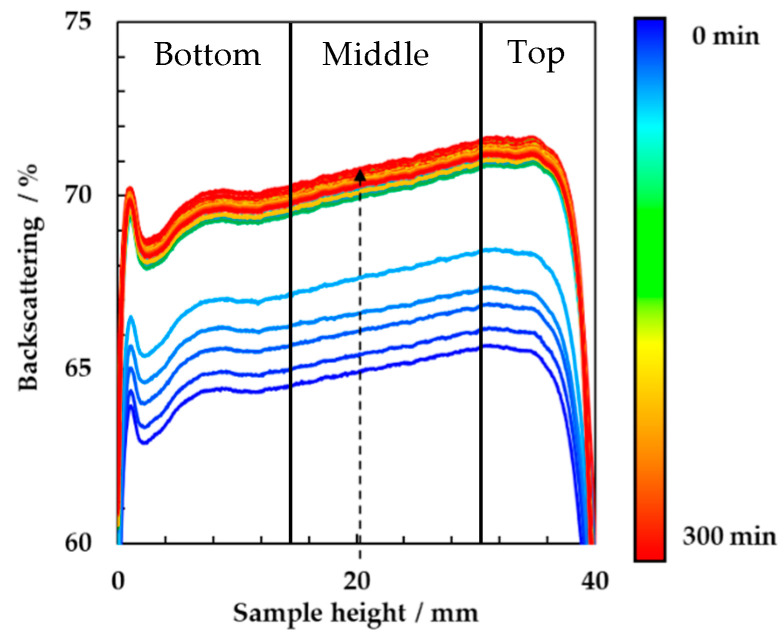
Backscattering spectra of milk sample incubated at 44 °C and inoculated with 4.5% (*w/w*) of yogurt starter culture for 1080 min.

Milk samples were prepared in glass tubes, inoculated with 4.5% (*w/w*) microbial starter culture and incubated at 44 °C inside the Turbiscan tower. The fermentation was monitored in real-time by multiple light scattering to obtain insights into the gelation process during the yogurt formation. The transient backscattering signal was acquired along the sample height (from 0 to 40 mm) as a function of the incubation time up to 300 min. In Figure 1, the backscattering signals have been reported. The recorded spectra were divided into three parts corresponding to the bottom, middle and top part of the sample´s height, where, respectively, sedimentation, aggregation and creaming phenomena prevailed. The backscattering values were lower at the bottom part of the tube (62%) and increased along the overall height (72%). This slope highlighted the occurrence of creaming. Also, the backscattering value increased over time, constantly along the sample height. This was due to the proteins network formation as result of the gelification occurring during milk fermentation. Along the time, the changes in backscattering became less pronounced and the monitoring was stopped within 5 h.

**Figure 2:**
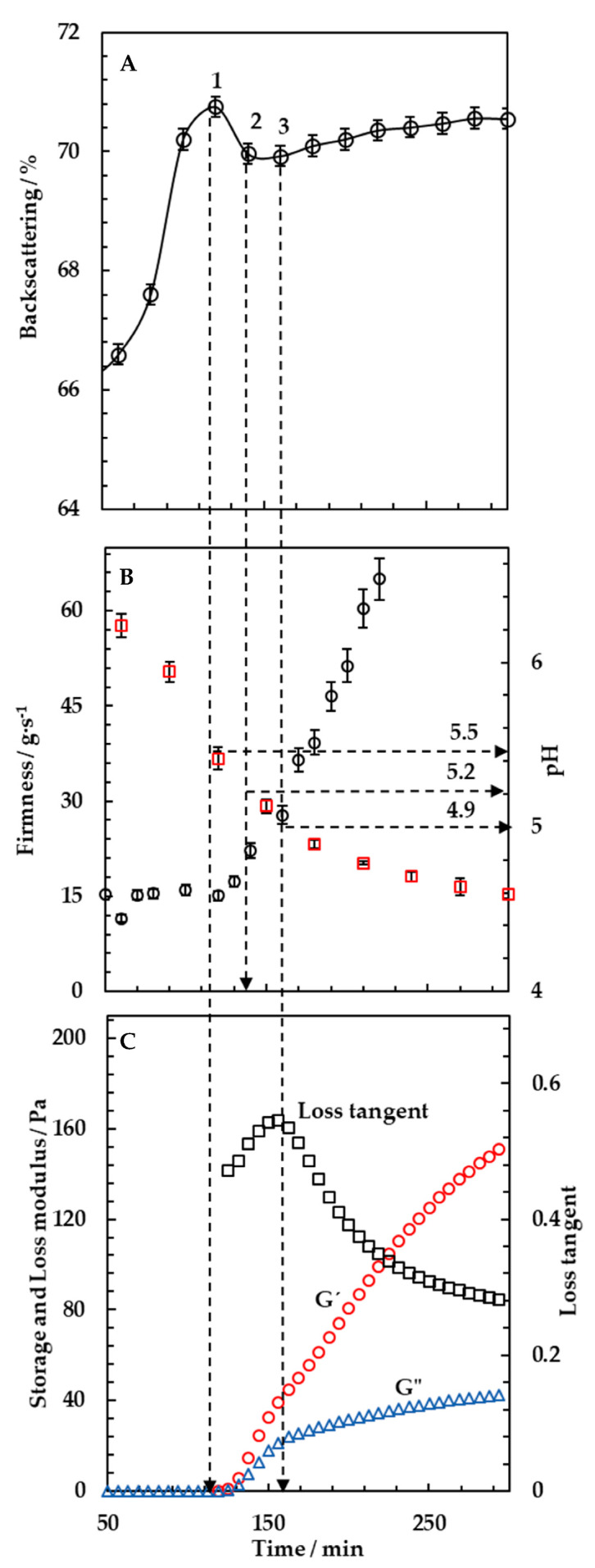
Changes of backscattering spectra during 1080 min measured in the middle height of the vial (20 mm), obtained from the analysis of backscattering data (**A**). Firmness, pH (**B**) and rheological properties (**C**) monitoring as a function of time for milk fermentation with 4.5% (*w/w*) of yogurt starter culture and an incubation temperature of 44 °C. Results are mean values of three replicates.

To better understand the phenomena detected by multiple light scattering during milk fermentation, the changes of the backscattering intensity in the middle part of the tube (at a sample height equal to 20 mm) were reported as a function of the time over a period of 300 min. The results are shown in Figure 2A. The first part of the curve was characterized by an exponential increase of the backscattering signal, which reached a maximum peak at 120 min (*n* = 3, M = 122, SD = 4). This increase was associated to the coagulation of milk proteins that induced a backscattering of light different from the surrounding medium. In this sample, the maximum value of backscattering (Figure 2A point 1) occurred at a pH of 5.5 (Figure 2B). Then, the backscattering slightly decreased (Figure 2A, point 2) and started to raise up again at pH equal to 5.2 (Figure 2A, point 3). The presence of these distinctive events indicated that the yogurt coagulation process proceeded based on different stages.

At a pH around 5.5, the backscattering signal had a maximum increase as can be seen in Figure 2A (point 1) and B. At this step, an intermediate network constituted by aggregates of soluble complexes in the serum was formed [23]. This intermediate network was not detectable in rheological and texture monitoring experiments, as shown in Figure 2B,C, where the value of the resulting firmness was equal to 15 ± 1.2 g·s^−1^ (same value of milk before the fermentation started) and the values of G’ and G″ were lower than 0.5 Pa. Once the preliminary protein network was formed, the pH continued to decrease, approaching the isoelectric point of caseins bound to whey proteins (~5.2). At this point, there was probably a decrease in the net negative charge and in the electrostatic repulsions between the casein particles led to a slight loosening of the network as shown by the decrease of the backscattering signal. Then, the attraction due to electrostatic interactions increased, causing micelles aggregation [24]. This was detected as a further raising of the backscattering signal. From this point, the behavior of the backscattering reflected the increase of the firmness values from 15 ± 1.2 to 25 ± 1.5 g·s^−1^ and the increase of G’ that exceeded 1 Pa. As also reported by Lucey [25], the phenomenon was associated to the formation of additional bonds between casein particles, the reorganization of the protein network and the incorporation of new free strands.

In this study, the denatured whey proteins were able to initiate the gelation at high pH values (5.5) close to the isoelectric point of β-lactoglobulin. Afterwards, the gelation was governed by casein–casein interactions as acidification continued [5,26]. It is known that the isoelectric points of proteins, constituting the milk aggregates, are equal to those of β-lactoglobulin (5.5), κ-casein (5.2) and α-lactalbumin (4.8). This is the reason why it was expected that there would be an early aggregation of β-lactoglobulin at pH 5.5 followed by a second aggregation of caseins at a lower pH [27].

This result was in agreement with previous studies where milk fermentation was also monitored by optical methods. Arango et al. [14] used a fiber optic sensor able to monitor a near infrared light backscattered during yogurt incubation. They observed that the first derivative of the light backscatter profile presented a peak occurring at the isoelectric point of β-lactoglobulin and another peak at a lower value corresponding to the one of κ-casein. On a similar vein, Alexander and Dalgleish [23] used transmission diffusing wave spectroscopy to study the gel formation of skim milk. They showed that the size of the casein micelles increased steadily but relatively slowly until reaching the isoelectric point of β-lactoglobulin. After this point, there was a sharp increase in size, which continued up to the isoelectric point of κ-casein, where a continuous gel started to form. In another study with a similar methodology, the results showed that the aggregation of casein micelles started around a pH of 5.5, after which an extensive aggregation occurred, continuing until a pH of around 5.0 [28].

In Figure 2C, a maximum in loss tangent was also observed. This behavior was interpreted as a partial loosening of bonds within and between casein molecules in the gel networks due to the solubilization of colloidal calcium phosphate and the release of Ca^2+^ at pH values lower than 5.8. The phenomenon of colloidal calcium phosphate solubilization appeared to alter the balance between the viscous and elastic components in the network that occurred at pH values around 4.9. This behavior was already reported in several studies [14,29]. However, the gel network remained intact due to the presence of stronger hydrophobic and electrostatic interactions as observed from multiple light scattering signals.

The analysis of the backscattering curve allowed to conclude that the time at which the first peak of the backscattering signal was detected could be identified as the starting point of the gel formation process. This was defined as gelation time.

### 3.2. Online Monitoring of the Effect of Starter Concentrations and Incubation Temperature on Milk Fermentation

**Figure 3:**
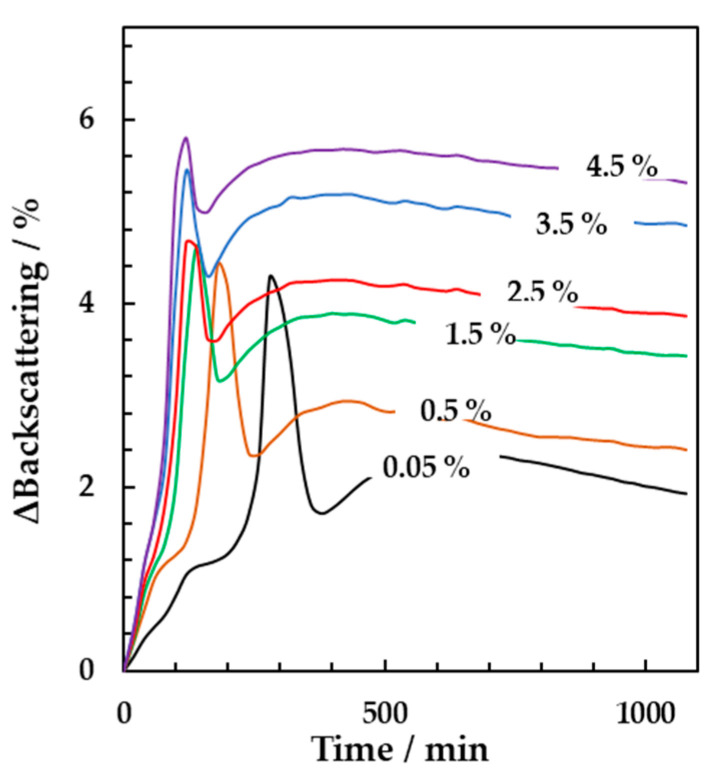
Backscattering signals acquired over 1080 min in the middle of the vial (height equal to 20 mm) at incubation temperature of 44 °C. The signals were obtained at different concentrations of yogurt starter culture from 0.05% to 4.5% (*w/w*). Results are mean values of triplicates.

Multiple light scattering was applied to study the effect of starter concentrations and incubation temperatures on the kinetics of yogurt gel formation. Milk samples were inoculated with different concentrations of starter up to 4.5% (*w/w*). The samples were incubated at 44 °C and the backscattering spectra were acquired over time. Figure 3 shows the resulting delta backscattering signals (calculated as the difference between the values obtained at a defined incubation time and those acquired at time zero) as a function of time. Each signal was obtained at a defined starter concentration. As expected, higher starter concentrations led to significantly higher backscattering intensities, which reached the maximum values for shorter times (Tukey, *n* = 3, *p* < 0.001). In detail, with a starter concentration of 0.5% (*w/w*) the gelation time resulted equal to 182 ± 5 min and decreased to 120 ± 4 min by adding 4.5% (*w/w*) of starter.

**Figure 4:**
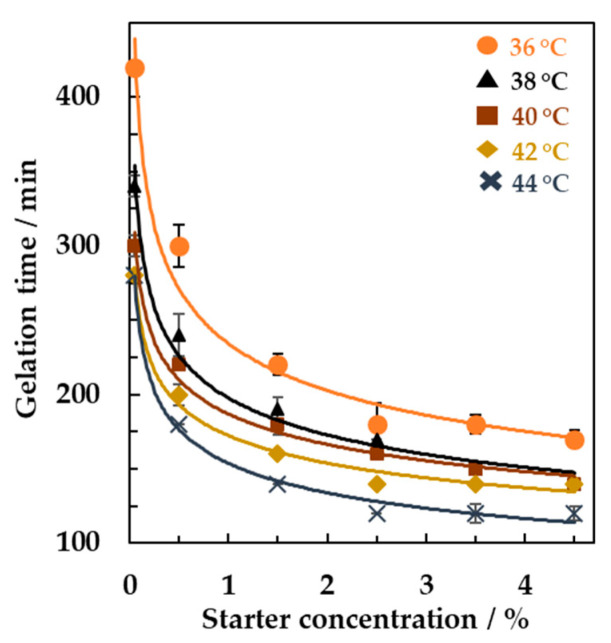
Gelation time obtained from backscattering signals versus different concentrations of yogurt starter as a function of the incubation temperature.

Multiple light scattering techniques were next tested to study the effect of the incubation temperature on the milk fermentation. The results are reported in Figure 4, where the gelation times of milk with the addition of different starter concentrations (from 0.05% to 4.5% *w/w*) were plotted as a function of different incubation temperatures (from 36 to 44 °C). The value of the gelation times was obtained from the analysis of the backscattering signals as a function of the incubation time obtained in the middle part of the vial for each starter concentration and incubation temperature. For all the concentration tested, an increase of the incubation temperature led to a significant decrease of the gelation time (Tukey, *n* = 3, *p* < 0.001). In detail, with a starter concentration equal to 1.5% (*w/w*) the resulting gelation time was equal to 230 ± 8 min at 36 °C and decreased to 143 ± 6 min at 44 °C. Within the range of temperature and starter concentration tested in the present study, the fastest gelation point was obtained by employing the incubation temperature of 44 °C and the starter concentration of 4.5% (*w/w*).

De Brabandere and De Baerdemaeker [21] also studied the effect of the incubation temperature by measuring the pH of the samples. They found that the fastest gelation occurred when an incubation temperature of 43.5 °C was used, similarly to what was obtained in this study by the analysis of multiple light scattering signals.

**Figure 5:**
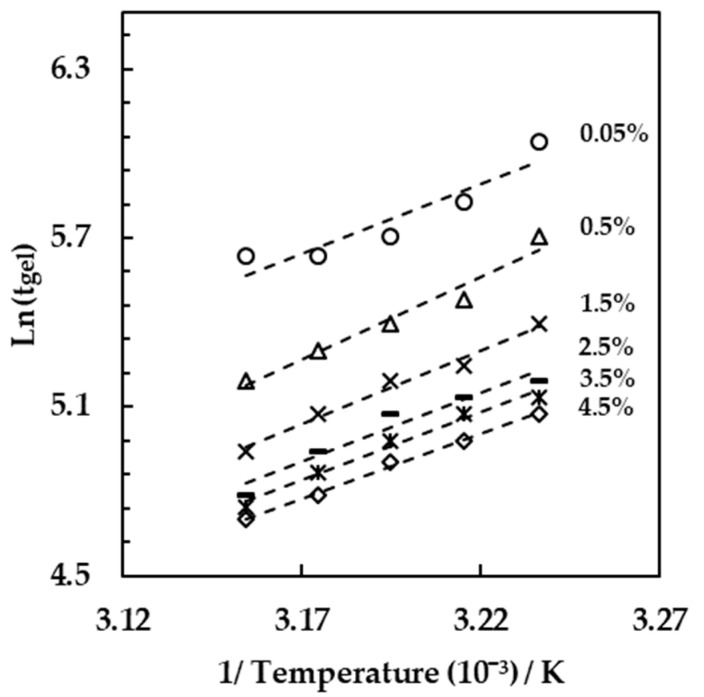
Natural log of the gel time plotted vs. the inverse of the incubation temperature.

With the gelation times obtained from the backscattering signals, an analysis of the gelation kinetic was performed using the time of gelation obtained at different temperatures for each starter concentration. The following equation was used:

(3) $$t_{gel}=A*e^{\frac{E_{gel}}{RT}}$$

where $t_{gel}$ is the gelation time from the backscattering signals, *A* is a constant, $E_{gel}$ is the gelation activation energy, *R* is the universal gas constant (J·mol^−1^·K^−1^), and *T* is the incubation temperature. Figure 5 shows the linearized form of the equation:

For each starter culture, the experimental points were fitted with a linear model with R^2^ ranging from 0.86 when the 0.05% (*w/w*) starter culture was used, to 0.99 with a 4.5% (*w/w*) starter concentration. The slope of the linear fit of each resulting curve was equal to E_gel_/R and allowed the resulting calculation of the mean value of the activation energy, which was equal to 42.21 ± 3.81 kJ/mol.

### 3.3. Online Monitoring of the Effect of Antibiotic on Milk Fermentation

**Figure 6:**
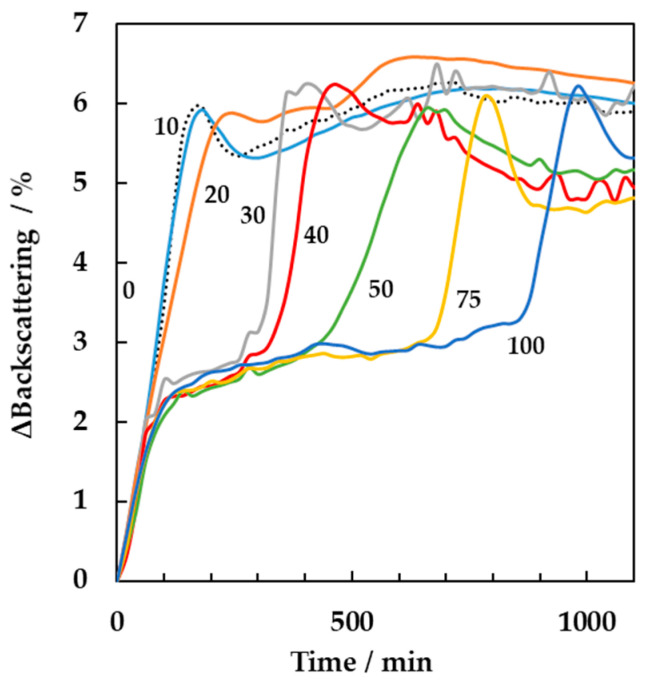
Backscattering spectra over time in absence and presence of ceftiofur antibiotic at concentrations equal to 0, 10, 20, 30, 40, 50, 75 and 100 µg/kg. The fermentation was performed at the incubation temperature of 44 °C with 4.5% (*w/w*) starter concentration. Results are mean values of triplicates.

Finally, the effect of the addition of ceftiofur antibiotic on the yogurt gel formation was studied by multiple light scattering. To this purpose, milk samples were spiked with ceftiofur in the range from 10 to 100 µg per kg of milk. The samples were then inoculated with 4.5% (*w/w*) starter concentration and incubated at 44 °C in the Turbiscan tower. Figure 6 shows the changes in backscattering signals acquired over time. The results indicated a significant delay (Tukey, *n* = 3, *p* < 0.001) of the gelation time when the milk was spiked with increasing concentrations of the antibiotic. The delay was directly proportional to the antibiotic concentrations as shown by the plateau region occurring in the time course of the backscattering signal prior to the detection of the gelation peak for milk samples containing from 30 to 100 μg/kg of antibiotic.

The gelation time was also linearly correlated with ceftiofur concentration, obtaining the following equation: y = 8.61*x − 142.6 (R^2^ = 0.97, sensitivity at 20 µg/kg = 1.7, limit of detection = 1.3 µg/kg) where y was the gelation time and x the antibiotic concentration. Few studies have been carried out to assess the effect of ceftiofur on fermented milks. A 50% reduction of lactic acid production with a difference in the pH value of 0.5 units was detected by Suhren [30], when the antibiotic concentrations added to yogurt were within 8 and 20 µg/kg. Similar findings were reported by Berruga et al. [31]. They showed that when ceftiofur (50, 100 and 150 μg/kg) was added to ewes’ milk, the acidification was significantly delayed for all concentrations assayed. Reybroeck [32] also noted diminished yogurt viscosity when milk came from ceftiofur treated animals. In all these studies, the applied methodologies were able to detect the effect of the antibiotic presence at concentrations close to the maximum residue level (100 µg/kg). However, they failed to provide results when much lower concentrations of antibiotic were present. The results reported in this study provides evidence of the potential of multiple light scattering technique to detect the effect of ceftiofur presence at very low concentrations far below the maximum residue level. Moreover, the technique allowed a direct, fast and online monitoring, avoiding the need of sampling procedures necessary when carrying out pH, texture, rheological or viscosity measurements. As a consequence, multiple light scattering can be considered as a valuable and reliable tool for the online monitoring and detection at the early stage of the presence of antibiotics in milk destined to fermented products.

## 4. Conclusions

In this study, multiple light scattering was tested for online monitoring yogurt gel formation. The technique was successfully applied to assess the effect of the processing parameters such as incubation temperature and concentration of yogurt starter during the fermentation process. The analysis of the firmness, pH and rheology properties confirmed the results obtained by multiple light scattering. The technique was also able to detect the effect of low traces of ceftiofur antibiotic during milk fermentation.

In conclusion, the application of multiple light scattering allowed a real time monitoring of the effect of the processing parameters on the development of yogurt gel formation, assessing its robustness in the field of milk fermentation.

## Data Availability

The study did not report any data.
